# Clinical Presentation, Grading, and Treatment Outcomes of Temporal Arteriovenous Malformations: A Systematic Review and Meta-Analysis

**DOI:** 10.7759/cureus.108804

**Published:** 2026-05-13

**Authors:** Ali K. Al-Shalchy, Nooruldeen H Ali Al-Khafaji

**Affiliations:** 1 Surgery, College of Medicine, University of Baghdad, Baghdad, IRQ

**Keywords:** clinical presentation, cognitive damage, neurological complications, spetzler–martin grades, temporal arteriovenous malformations

## Abstract

Temporal arteriovenous malformations (AVMs) represent a specific subset of intracranial AVMs often linked to eloquent cortical regions and seizure risk. Their clinical features and treatment results are not fully understood, especially regarding location-specific factors. This systematic review and meta-analysis followed Preferred Reporting Items for Systematic Reviews and Meta-Analyses (PRISMA) guidelines, involving a comprehensive search of PubMed and Scopus for studies on temporal AVMs with available clinical and outcome data. Only studies focusing on lesions within the temporal lobe were included. Data collected encompassed patient demographics, clinical presentation, Spetzler-Martin grades, treatment methods, and outcomes, with pooled estimates computed via a random-effects model. Seventeen studies with 319 patients were analyzed. The average age was 38.0 years, with sex distribution being relatively balanced, with males representing 147/319 patients (46.1%) and a pooled male proportion of 51.5%. The most common presentations were hemorrhage (147/319, 46.1%) and seizures (115/319, 36.1%). Low-grade lesions (Spetzler-Martin I-II) were most frequent, followed by intermediate and high-grade types. Surgical resection was the primary treatment approach. Favorable functional outcomes (modified Rankin Scale ≤ 2 or good Glasgow Outcome Scale) occurred in 235 patients (73.7%), while 65 (20.4%) had poor outcomes. Mortality was low, at 10 patients (3.1%). Visual deficits were reported in 28 patients (8.8%), and permanent neurological deficits in 12 (3.8%). Recurrence was rare, observed in five patients (1.6%), with 10 patients (3.1%) needing reoperation. Temporal AVMs have a distinctive clinical profile, mainly involving seizures and hemorrhage, with microsurgical resection being the main treatment modality that yields high rates of favorable outcomes with acceptable morbidity. Adjunctive therapies like endovascular embolization and radiosurgery are used selectively based on specific clinical situations. Outcomes are affected by lesion features and treatment strategies, highlighting the importance of personalized management and the need for further high-quality research.

## Introduction and background

Brain arteriovenous malformations (AVMs) are rare vascular anomalies characterized by direct arteriovenous shunting without an intervening capillary bed. This condition poses a lifelong risk of intracranial hemorrhage, seizures, and neurological complications. Their estimated prevalence is about 10-18 per 100,000 adults, with a natural history marked by a sustained annual hemorrhage risk generally estimated at 2%-4%, which increases after rupture [1,2]. Temporal AVMs form a distinct subgroup of intracranial AVMs, representing roughly 12%-16% of cases in previous series. They are clinically significant because the temporal lobe plays a crucial role in language, memory, vision, and epileptogenesis. Consequently, these lesions often present with seizures, although hemorrhage remains a common presentation [3,4].

Managing temporal AVMs remains difficult because treatment must carefully balance complete removal with the risk of neurological and cognitive damage. Modern management strategies are multimodal, combining microsurgical resection, endovascular embolization, and stereotactic radiosurgery based on lesion anatomy and patient-specific factors [3]. In surgical studies focusing on temporal AVMs, many lesions are low- to moderate-grade and thus deemed suitable for resection in selected cases, though outcomes are still affected by factors such as lesion subtype, eloquence, venous drainage, and the treatment method [4,5]. Since most literature consists of single-center reports and mixed AVM groups, the specific presentation and combined outcomes of temporal AVMs as a distinct category are not fully established. Therefore, this systematic review and meta-analysis aims to compile the existing evidence on temporal AVMs, focusing on epidemiology, clinical features, grading patterns, and treatment results.

## Review

Methods

Study Design and Search Strategy

This systematic review and meta-analysis adhered to the Preferred Reporting Items for Systematic Reviews and Meta-Analyses (PRISMA) guidelines [6]. A thorough literature search was conducted in PubMed and Scopus from their beginnings up to the final search date. A comprehensive literature search was performed in PubMed and Scopus from database inception to February 28, 2026. The search strategy combined terms related to AVMs and temporal/hippocampal location. The PubMed search was conducted using the following strategy: (("Arteriovenous Malformations"[Mesh] OR arteriovenous malformation OR AVM OR cerebral AVM* OR brain AVM* OR intracranial AVM*) AND ("Hippocampus"[Mesh] OR hippocamp* OR mesial temporal OR medial temporal)). The Scopus search was conducted using the following strategy: (AVM OR arteriovenous malformation) AND (hippocamp* OR temporal). Reference lists of included studies were also manually screened to identify additional relevant articles.

Study Selection

All retrieved records were imported into a reference management system, with duplicates removed prior to screening. Two reviewers (N.H.A.A and A.K.A.) independently screened titles and abstracts based on predefined eligibility criteria. The same reviewers then independently assessed full-text articles. Any disagreements were resolved through discussion between the reviewers.

Eligibility Criteria

Studies were included if they provided clinical data on patients with AVMs limited to the temporal lobe, including details on presentation, grading, treatment, or outcomes. Both retrospective and prospective cohort studies, as well as case series, qualified for inclusion. Studies were excluded if they involved AVMs outside the temporal lobe, were from mixed-location cohorts without separate data for the temporal lobe, were review articles or editorials, or lacked enough clinical detail. When multiple publications reported on the same cohort, the most comprehensive or recent study was chosen.

Full-Text Exclusion

Thirty full-text articles were evaluated for eligibility, with 13 excluded for various reasons. These reasons included lack of extractable temporal AVM-specific data, mixed AVM cohorts where temporal lesions could not be distinguished from other locations, non-AVM pathologies or combined vascular malformation groups without separable AVM data, inadequate outcome reporting, and overlapping or duplicate patient populations.

Data Extraction

Data extraction was performed independently by two reviewers (N.H.A.A. and A.K.A.) using a standardized extraction sheet developed before analysis. Extracted variables included study characteristics, country, study design, number of temporal AVM patients, age, sex, rupture status, seizure presentation, headache, neurological deficit, AVM size, Spetzler-Martin grade, treatment modality, complete resection, complete obliteration, residual AVM, recurrence, reoperation, complications, mortality, and functional outcome. The Spetzler-Martin grading system classifies AVMs according to nidus size, eloquence of adjacent brain, and venous drainage pattern. Scores range from grade I to V, with higher grades indicating greater expected surgical complexity and operative risk. Functional outcomes were extracted as reported by each study. When available, a favorable outcome was defined as modified Rankin Scale (mRS) ≤ 2. For studies reporting the Glasgow Outcome Scale (GOS), a favorable outcome was defined as GOS 4-5, corresponding to moderate disability or good recovery, whereas a poor outcome was defined as GOS 1-3. Because mRS and GOS are not fully equivalent, these outcomes were harmonized only into broad favorable versus unfavorable categories for pooled descriptive analysis. Follow-up duration was extracted for each study when reported and is provided in the study characteristics table. Any discrepancies were resolved through discussion and consensus.

Risk of Bias Assessment

The methodological quality of the included studies was assessed using the Risk Of Bias In Non-randomized Studies of Interventions (ROBINS-I) tool for cohort studies and the Joanna Briggs Institute (JBI) checklist for the single case report [7,8]. Discrepancies in risk of bias judgments were resolved by discussion. The included studies showed an overall moderate-to-serious risk of bias, mainly because of their retrospective design and the selection of cohorts treated surgically. The primary sources of bias were confounding factors and participant selection, especially in older studies, whereas the classification of interventions was generally considered low risk, as illustrated in Table 1 [4,5,9-22].

**Table 1:** ROBINS-I risk of bias assessment ROBINS-1: Risk Of Bias In Non-randomized Studies of Interventions

Study	Confounding	Selection	Intervention classification	Deviations	Missing data	Outcome measurement	Reporting	Overall
Clarençon et al., 2023 [9]	Moderate	Moderate	Low	Low	Moderate	Low	Moderate	Moderate
Scherschinski et al., 2023 [5]	Moderate	Moderate	Low	Low	Low	Low	Moderate	Moderate
Mandel et al., 2022 [10]	Moderate	Moderate	Low	Low	Moderate	Moderate	Moderate	Moderate
Tao et al., 2022 [11]	Moderate	Moderate	Low	Low	Moderate	Moderate	Moderate	Moderate
Lopez-Ojeda et al., 2013 [12]	Serious	Moderate	Low	Low	Moderate	Moderate	Moderate	Serious
de Oliveira et al., 2012 [13]	Moderate	Moderate	Low	Low	Moderate	Moderate	Moderate	Moderate
Lv et al., 2010 [14]	Moderate	Moderate	Low	Low	Moderate	Moderate	Moderate	Moderate
Canals et al., 2013 [4]	Serious	Moderate	Low	Low	Moderate	Moderate	Moderate	Serious
Weber et al., 2007 [15]	Moderate	Moderate	Low	Low	Low	Moderate	Moderate	Moderate
Nagata et al., 2006 [16]	Moderate	Moderate	Low	Low	Moderate	Moderate	Moderate	Moderate
Du et al., 2004 [17]	Serious	Moderate	Low	Low	Moderate	Moderate	Moderate	Serious
Iwama et al., 2003 [18]	Moderate	Moderate	Low	Low	Moderate	Moderate	Moderate	Moderate
Kikuchi et al., 1997 [19]	Serious	Moderate	Low	Low	Moderate	Moderate	Moderate	Serious
Malik et al., 1996 [20]	Serious	Moderate	Low	Low	Moderate	Moderate	Moderate	Serious
Yeh et al., 1993 [21]	Serious	Moderate	Low	Low	Moderate	Moderate	Moderate	Serious
Yeh et al., 1990 [22]	Serious	Moderate	Low	Low	Moderate	Moderate	Moderate	Serious

Risks associated with missing data, outcome measurement, and selective reporting were mainly moderate, due to variability in follow-up and how outcomes were defined. The only case report indicated a moderate risk of bias. Overall, these findings should be considered within the framework of observational data, which has inherent methodological limitations, as illustrated in Table 2 [23].

**Table 2:** Risk of bias assessment: JBI critical appraisal checklist for case reports JBI: Joanna Briggs Institute

Study	Demographics	History	Clinical condition	Diagnostics	Intervention	Post-intervention	Adverse events	Takeaway	Overall
Oka et al., 1990 [23]	Yes	Yes	Yes	Yes	Yes	Yes	Unclear	Yes	Moderate

Statistical Analysis

Meta-analyses were performed using jamovi version 2.7 and R version 4.5, with analyses supported by the metafor package. Pooled estimates for continuous variables were calculated using random-effects models and reported as means with 95% confidence intervals (CIs). For proportional outcomes, random-effects meta-analyses were performed using an appropriate transformation for proportion data to ensure that pooled estimates and CIs remained within the valid 0%-100% range. Pooled estimates were back-transformed and reported as percentages with 95% CIs. Between-study heterogeneity was assessed using the I² statistic. The between-study variance estimator, continuity correction, and handling of zero-event studies were specified according to the model output. Software and package references were included to support reproducibility [24-26].

Results

Study Characteristics

**Figure 1:**
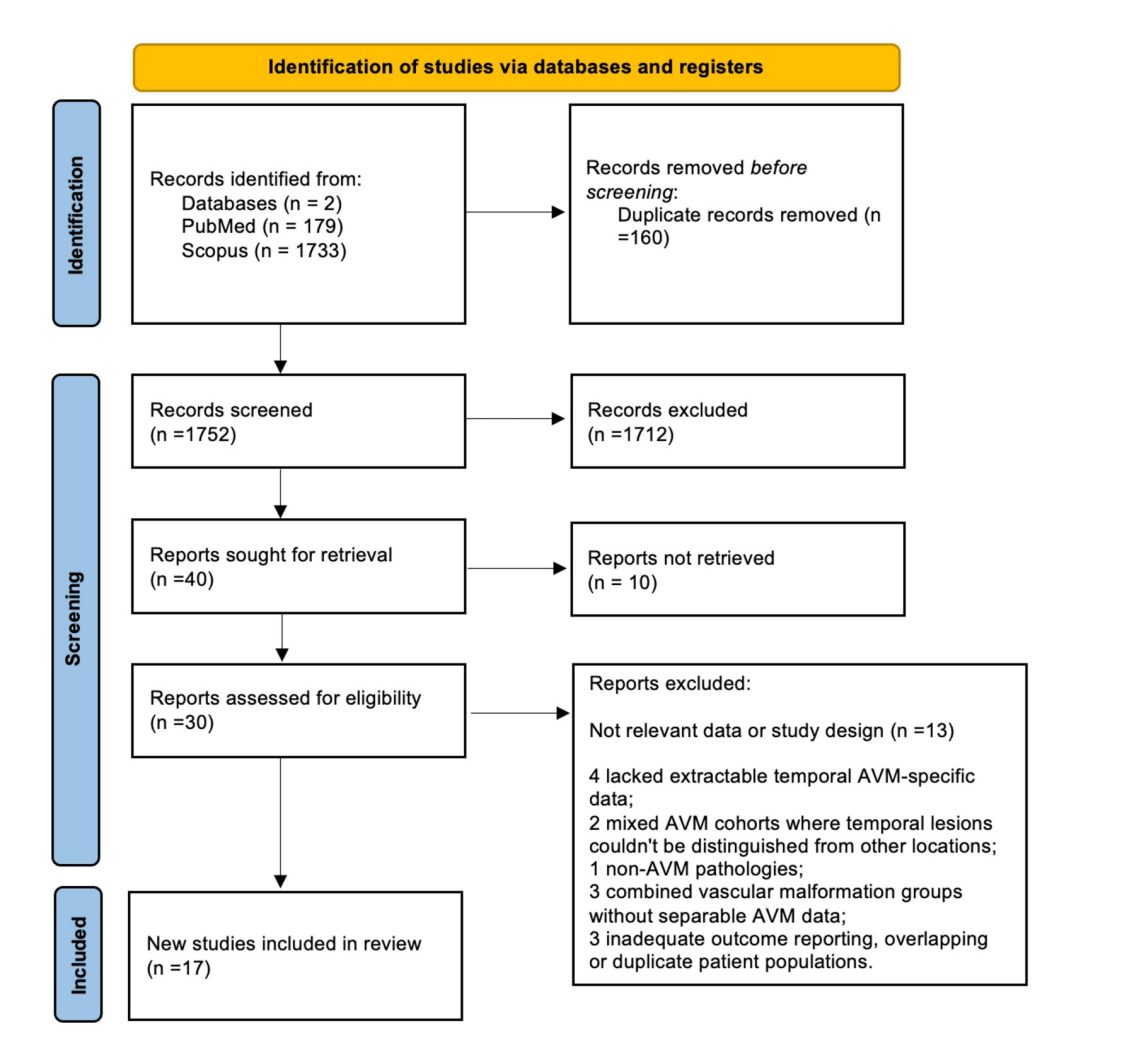
PRISMA flow diagram PRISMA: Preferred Reporting Items for Systematic Reviews and Meta-Analyses; AVM: arteriovenous malformation

A total of 1,912 records were identified through database searching. After reviewing full texts, only 17 studies were included in the final analysis (Figure 1). These studies, conducted between 1990 and 2023, mainly consisted of retrospective case series focusing on temporal AVMs. Most patients across these studies received microsurgical treatment, with few cases involving non-surgical or standalone alternative methods. The cohorts varied widely in sample size, lesion features, and outcome reporting, reflecting differences in study design and institutional practices. Despite this heterogeneity, all studies provided data specific to temporal AVMs, allowing for combined analysis of clinical presentation, grading, management, and outcomes as illustrated in Tables 3, 4 [4,5,9-23].

**Table 3:** Patients and AVM characteristics SM: Spetzler-Martin arteriovenous malformation grading system; NR: not reported; AVM: arteriovenous malformation

Author, year	Country	Study design	Total N	Age mean	Age SD	Male N	Male percentage	Ruptured N	Ruptured percentage	Unruptured N	Unruptured percentage	Seizure N	Hemorrhage N	Headache N	Neurodeficit N	AVM size mean (cm)	AVM size SD (cm)	SM I-II N	SM I-II percentage	SM III N	SM III percentage	SM IV-V N	SM IV-V percentage	Associated vascular anomaly N
Du et al., 2004 [17]	USA	Retrospective case series	10	23.7	19.0	4	40.0%	6	60.0%	4	40.0%	3	6	1	1	2.5	12.3	7	70.0%	1	10.0%	2	20.0%	0
Kikuchi et al., 1997 [19]	Japan	Retrospective case series	9	37.1	14.6	7	77.8%	7	77.8%	2	22.2%	0	7	0	0	NR	NR	NR	NR	NR	NR	NR	NR	NR
Canals et al., 2013 [4]	USA	Retrospective case series	88	39.0	NR	42	47.7%	45	51.1%	43	48.9%	24	45	15	0	2.7	NR	54	61.4%	25	28.4%	9	10.2%	NR
Lopez-Ojeda et al., 2013 [12]	Canada	Retrospective case series	29	39.2	16.0	10	34.5%	15	51.7%	14	48.3%	12	15	2	4	NR	NR	21	72.4%	8	27.6%	0	0.0%	NR
Malik et al., 1996 [20]	USA	Retrospective case series	24	33.0	14.6	11	45.8%	7	29.2%	17	70.8%	11	7	4	13	3.9	1.5	NR	NR	NR	NR	NR	NR	1
Nagata et al., 2006 [16]	Japan	Retrospective case series	26	34.2	15.1	15	57.7%	22	84.6%	4	15.4%	12	22	0	17	NR	NR	14	53.8%	12	46.2%	0	0.0%	NR
Scherschinski et al., 2023 [5]	USA	Retrospective comparative cohort	59	31.0	18.0	30	50.8%	34	57.6%	25	42.4%	0	34	0	0	NR	NR	24	40.7%	25	42.4%	7	11.9%	NR
de Oliveira et al., 2012 [13]	Brazil	Retrospective case series	3	38.7	2.1	0	0.0%	1	33.3%	2	66.7%	2	1	0	0	NR	NR	NR	NR	NR	NR	NR	NR	NR
Yeh et al., 1990 [22]	USA	Retrospective case series	12	36.3	NR	8	66.7%	0	0.0%	12	100.0%	12	0	0	0	NR	NR	NR	NR	NR	NR	NR	NR	NR
Yeh et al., 1993 [21]	USA	Prospective case series	17	NR	NR	NR	NR	0	0.0%	17	100.0%	17	0	0	0	NR	NR	NR	NR	NR	NR	NR	NR	NR
Mandel et al., 2022 [10]	Brazil	Retrospective case series	12	33.5	NR	5	41.7%	3	25.0%	9	75.0%	0	3	0	0	3.2	NR	9	75.0%	2	16.7%	1	8.3%	NR
Lv et al., 2010 [14]	China	Retrospective case series	7	33.3	11.2	6	85.7%	0	0.0%	7	100.0%	7	0	0	0	3.9	1.1	NR	NR	NR	NR	NR	NR	NR
Tao et al., 2022 [11]	China	Retrospective case series	4	48.8	3.7	3	75.0%	0	0.0%	4	100.0%	3	0	0	1	58.9	16.3	0	0.0%	2	50.0%	2	50.0%	NR
Clarençon et al., 2023 [9]	France	Retrospective case series	4	NR	NR	NR	NR	1	25%	3	75%	4	0	0	0	35.5	23.7	2	50%	1	25%	1	25%	NR
Weber et al., 2007 [15]	Germany	Retrospective case series	9	36.8	15.8	2	50%	9	100%	0	0	6	1	2	0	34.7	14	5	55.6	2	22.2	2	22.2	NR
Iwama et al., 2003 [18]	Switzerland	Retrospective case series	3	58.7	4.2	1	33.4%	3	100%	0	0	2	3	0	1	4	1	NR	NR	NR	NR	NR	NR	NR
Oka et al., 1990 [23]	Japan	Case report	3	31.3	20.2	3	100%	3	100%	0	0	0	3	2	2	NR	NR	NR	NR	NR	NR	NR	NR	NR

**Table 4:** Treatment and outcomes GOS: Glasgow Outcome Score; NR: not reported; AVM: arteriovenous malformation; mRS: modified Rankin Scale

Author, year	Surgery N	Surgery percentage	Embolization N	Embolization percentage	Radiosurgery N	Radiosurgery percentage	Complete resection N	Complete resection percentage	Partial resection N	Partial resection percentage	Cortical excision N	Cortical excision percentage	Complete obliteration N	Complete obliteration percentage	Residual AVM N	Residual AVM percentage	Complication total N	Complication total percentage	Permanent deficit N	Hemiparesis N	Aphasia N	Visual deficit N	Hemorrhage postop N	Recurrence N	Recurrence percentage	Reoperation N	Reoperation percentage	Mortality N	Mortality percentage	mRS ≤ 2 or GOS good N	mRS ≤ 2 or GOS good percentage	mRS > 2 or GOS poor N	mRS > 2 or GOS poor percentage	Follow-up mean months
Du et al., 2004 [17]	10	100.0%	0	0.0%	1	10.0%	9	90.0%	1	10.0%	0	0.0%	9	90.0%	1	10.0%	3	30.0%	0	1	2	0	0	0	0.0%	0	0.0%	0	0.0%	6	60.0%	4	40.0%	15.6
Kikuchi et al., 1997 [19]	9	100.0%	3	33.3%	0	0.0%	9	100.0%	0	0.0%	NR	NR	9	100.0%	0	0.0%	3	33.3%	0	3	0	0	0	0	0.0%	0	0.0%	0	0.0%	5	55.6%	4	44.4%	NR
Canals et al., 2013 [4]	88	100.0%	59	67.0%	6	6.8%	82	93.2%	6	6.8%	NR	NR	82	93.2%	6	6.8%	19	21.6%	NR	NR	NR	NR	3	0	0.0%	4	4.5%	4	4.5%	71	87.0%	11	13.0%	NR
Lopez-Ojeda et al., 2013 [12]	29	100.0%	15	51.7%	1	3.4%	27	93.1%	2	6.9%	NR	NR	27	93.1%	2	6.9%	7	24.1%	3	3	0	8	0	1	3.4%	2	6.9%	0	0.0%	25	86.2%	4	13.8%	NR
Malik et al., 1996 [20]	24	100.0%	7	29.2%	0	0.0%	23	95.8%	1	4.2%	NR	NR	23	95.8%	1	4.2%	6	25.0%	3	2	3	4	2	1	4.2%	1	4.2%	1	4.2%	10	41.7%	14	58.3%	27.0
Nagata et al., 2006 [16]	26	100.0%	6	23.1%	0	0.0%	26	100.0%	0	0.0%	NR	NR	26	100.0%	0	0.0%	18	69.2%	0	0	0	16	0	0	0.0%	0	0.0%	0	0.0%	10	38.5%	16	61.5%	NR
Scherschinski et al., 2023 [5]	59	100.0%	38	64.4%	0	0.0%	NR	NR	NR	NR	39	66.1%	53	89.80%	6	10.2%	17	28.8%	NR	NR	NR	NR	NR	NR	NR	NR	NR	2	3.4%	52	88.1%	7	11.9%	22, 29
de Oliveira et al., 2012 [13]	3	100.0%	0	0.0%	0	0.0%	3	100.0%	0	0.0%	0	0.0%	3	100.0%	0	0.0%	0	0.0%	0	0	0	0	0	0	0.0%	0	0.0%	0	0.0%	3	100.0%	0	0.0%	140.3
Yeh et al., 1990 [22]	12	100.0%	0	0.0%	0	0.0%	12	100.0%	0	0.0%	10	83.3%	12	100.0%	0	0.0%	4	33.3%	3	0	0	0	0	1	8.3%	1	8.3%	0	0.0%	12	100.0%	0	0.0%	47.0
Yeh et al., 1993 [21]	17	100.0%	0	0.0%	0	0.0%	17	100.0%	0	0.0%	12	70.6%	17	100.0%	0	0.0%	2	11.8%	1	1	0	0	0	1	5.9%	1	5.9%	0	0.0%	16	94.1%	1	5.9%	57.6
Mandel et al., 2022 [10]	12	100.0%	2	16.7%	0	0.0%	NR	NR	NR	NR	NR	NR	12.0	100.0%	0.0%	0.0%	0.0%	0.0%	0	0	0	0	0	NR	NR	NR	NR	NR	NR	NR	NR	NR	NR	NR
Lv et al., 2010 [14]	0	0.0%	7	100.0%	0	0.0%	0	0.0%	0	0.0%	0	0.0%	1	14.3%	6	85.7%	0	0.0%	0	0	0	0	0	0	0.0%	0	0.0%	0	0.0%	7	100.0%	0	0.0%	87.6
Tao et al., 2022 [11]	4	100.0%	4	100.0%	0	0.0%	0	0.0%	0	0.0%	0	0.0%	4	100.0%	0	0.0%	0	0.0%	NR	NR	NR	NR	NR	NR	NR	NR	NR	0	0.0%	4	100.0%	0	0.0%	NR
Clarençon et al., 2023 [9]	4	100%	4	100%	0	0	NR	NR	NR	NR	NR	NR	4	100%	0	0	1	25%	0	1	0	0	1	0	0	0	0	0	0	4	100%	0	0	8
Weber et al., 2007 [15]	9	100%	9	100%	0	0	NR	NR	NR	NR	NR	NR	6	66.60%	3	33.40%	NR	NR	NR	NR	NR	NR	NR	NR	NR	NR	NR	0	0	8	88.9	0	0	12.3
Iwama et al., 2003 [18]	3	100%	0	0	0	0	2	66.7%	1	33.40%	NR	NR	NR	NR	NR	NR	NR	NR	NR	NR	NR	NR	NR	NR	NR	NR	NR	2	66.70%	1	33.40%	2	66.60%	12
Oka et al., 1990 [23]	3	100%	0	0	0	0	3	100%	0	0	NR	NR	3	100%	0	0	2	66.70%	2	2	1	0	0	1	33.4%	1	33.4%	1	33.4%	1	33.4%	2	66.7%	22

Clinical Characteristics and Presentation

Seventeen studies involving 319 patients were analyzed. The average age was 38.0 years (95% CI: 32.6-43.3; I² = 90.7%). The crude proportion of male patients was 147/319 (46.1%), while the pooled male proportion was 51.5% (95% CI: 41.7-61.2). Seizures were a common symptom, present in 50.4% of patients (95% CI: 30.0-70.9; I² = 96.6%), or 115 out of 319 (36.1%). Hemorrhagic presentation was reported in 46.4% of cases (95% CI: 30.0-62.7; I² = 92.2%), corresponding to 147 out of 319 patients (46.1%). Visual deficits occurred in 9.7% of cases (95% CI: −1.7-21.2; I² = 77.1%), or 28 out of 319 (8.8%) patients (Table 5).

**Table 5:** Clinical presentation and AVM characteristics pooled estimates CI: confidence interval; SM: Spetzler-Martin; AVM: arteriovenous malformation

Variable	k	Pooled estimate	95% CI	I² (%)
Age (years)	12	38.0	32.6-43.3	90.7
Male (%)	15	51.5	41.7-61.2	68.2
Seizures (%)	17	50.4	30.0-70.9	96.6
Hemorrhage (%)	17	46.4	30.0-62.7	92.2
Visual deficit (%)	12	9.7	-1.7-21.2	77.1
SM I-II (%)	9	54.8	42.8-66.8	72.3
SM III (%)	9	30.3	22.3-38.3	41.8
SM IV-V (%)	9	9.6	3.1-16.0	52.4
AVM size (cm)	7	6.83	4.26-9.40	94.1

AVM Characteristics

Most lesions were low-grade (Spetzler-Martin I-II), making up 54.8% (95% CI: 42.8-66.8; I² = 72.3%). Grade III lesions represented 30.3% (95% CI: 22.3-38.3; I² = 41.8%), while high-grade lesions (IV-V) were less common at 9.6% (95% CI: 3.1-16.0; I² = 52.4%). The average AVM size was 6.83 cm (95% CI: 4.26-9.40; I² = 94.1%).

Treatment Characteristics and Outcomes

Surgical management was the primary treatment, with a pooled proportion of 95.7% (95% CI: 89.6-100.0; I² = 78.2%). High rates of favorable functional outcomes were noted, with 76.5% of patients reaching mRS ≤ 2 or good GOS (95% CI: 66.0-86.9; I² = 80.8%), representing 235 out of 319 patients (73.7%). Poor outcomes (mRS > 2 or poor GOS) were observed in 22.2% (95% CI: 11.7-32.6; I² = 82.0%), or 65 out of 319 patients (20.4%). Mortality was low, with a pooled rate of 2.9% (95% CI: −0.3-6.0; I² = 8.5%), reflecting 10 of 319 patients (3.1%) (Table 6).

**Table 6:** Treatment outcomes and complications CI: confidence interval; I²: inconsistency statistic; mRS: modified Rankin Scale; GOS: Glasgow Outcome Scale; AVM: arteriovenous malformation

Variable	k	Pooled estimate	95% CI	I² (%)
Surgical treatment (%)	17	95.7	89.6-100.0	78.2
Good outcome (mRS ≤ 2/GOS good) (%)	16	76.5	66.0-86.9	80.8
Poor outcome (mRS > 2/GOS poor) (%)	16	22.2	11.7-32.6	82.0
Mortality (%)	16	2.9	-0.3-6.0	8.5
Neurological deficit (%)	17	13.3	5.3-21.3	84.8
Permanent deficit (%)	12	6.9	0.2-13.5	39.1
Residual AVM (%)	16	7.9	1.5-14.4	66.8
Partial resection (%)	13	4.3	0.7-7.9	0
Recurrence (%)	12	0.9	-1.6-3.4	0
Reoperation (%)	12	4.0	0.6-7.4	0

Complications and Recurrence

Overall, neurological deficits were observed in 13.3% of patients (95% CI: 5.3-21.3; I² = 84.8%), with permanent deficits in 6.9% (95% CI: 0.2-13.5; I² = 39.1%), representing 12 out of 319 patients (3.8%). Residual AVMs occurred in 7.9% of cases (95% CI: 1.5-14.4; I² = 66.8%), while partial resection was seen in 4.3% (95% CI: 0.7-7.9; I² = 0%). Recurrence was uncommon, with a pooled rate of 0.9% (95% CI: −1.6-3.4; I² = 0%), amounting to five out of 319 patients (1.6%), and reoperation was necessary in 4.0% of cases (95% CI: 0.6-7.4; I² = 0%), corresponding to 10 out of 319 patients (3.1%).

Discussion

This systematic review and meta-analysis focus on temporal AVMs, detailing their clinical features, anatomical characteristics, surgical options, and treatment outcomes. The aggregated data indicate an average age of 38.0 years, with a nearly equal gender split and a slight male dominance at 51.5%. Seizures and hemorrhages are common, seen in 50.4% and 46.4% of cases, respectively. This suggests that temporal AVMs are a distinct clinical subgroup, with cortical involvement raising both seizure risk and hemorrhagic potential. The considerable heterogeneity in age, seizure incidence, hemorrhage, and lesion size likely results from differences in referral patterns, treatment timeframes, inclusion criteria, and the composition of cohorts, whether they contain more ruptured, epileptic, or surgically managed patients.

This review found that most temporal AVMs were low- or moderate-grade lesions. Specifically, 54.8% of cases were Spetzler-Martin grade I-II, 30.3% were grade III, and 9.6% were grades IV-V. This grading distribution is significant because surgical risk for AVMs depends heavily on lesion size, eloquence, and venous drainage-factors that underlie the Spetzler-Martin system and its simplified Spetzler-Ponce version [27,28]. The higher prevalence of lower-grade lesions likely explains the higher rate of surgical interventions and the generally positive outcomes observed. Nonetheless, the average AVM size varied considerably, indicating that the temporal AVMs studied were not uniform in anatomy and probably included both more accessible lateral lesions and more complex medial or deep malformations.

Surgery was the dominant treatment modality in the available temporal AVM literature, with a pooled surgical treatment proportion of 95.7%. This finding should be interpreted as a description of the evidence base rather than proof of surgical superiority. Most included studies were surgical series, and therefore, the dataset is inherently enriched for patients considered suitable for operative management. Broader AVM literature similarly emphasizes that treatment selection is individualized and depends on rupture status, lesion grade, anatomy, age, clinical presentation, and institutional expertise [3,29]. Accordingly, the high surgical proportion in this review most likely reflects patient selection, lesion accessibility, and the historical centrality of microsurgery in temporal AVM management.

The pooled results indicate good treatment durability, with residual AVM present in 7.9%, partial resection in 4.3%, recurrence in 0.9%, and reoperation in 4.0%. These findings imply that definitive lesion control is frequently achievable based on published temporal AVM surgical data. However, these outcomes should be interpreted considering surgical selection criteria. Lower-grade, compact, and surgically accessible lesions are more often resected, whereas deeper or more eloquent temporal regions are managed more selectively. Thus, the pooled estimates reflect outcomes of reported treated groups rather than the entire range of temporal AVMs seen in clinical practice.

The role of embolization and radiosurgery in the literature on temporal AVMs included in this review is less clearly defined. Embolization is often used alongside surgery to reduce blood flow, devascularize the lesion, or manage difficult-to-treat feeders. In broader AVM treatment practices, embolization can support multimodal approaches but has its own risks and does not always achieve a complete cure when used alone [3,30]. Radiosurgery is typically reserved for smaller, deep, or high-risk AVMs for surgery, but obliteration takes time, and the risk of hemorrhage may continue during the latency period [31]. Since non-surgical treatments were underrepresented in the temporal AVM dataset, direct comparisons among microsurgery, embolization, radiosurgery, and conservative management are limited. Future research should stratify outcomes by treatment type and AVM subtype to better understand each approach's role.

Overall, functional outcomes were mostly positive, with 76.5% of patients reaching good outcome status, defined as mRS ≤ 2 or good GOS, while 22.2% experienced poor outcomes. Mortality was low at 2.9%, and permanent deficits were observed in 6.9%. These results indicate that treating selected temporal AVMs often leads to acceptable functional outcomes. However, neurological deficits were reported in 13.3%, and the high variability in neurological morbidity suggests that the risk differs significantly among different cohorts. This variation is clinically understandable, considering the complex functional anatomy of the temporal lobe, which includes language networks in the dominant hemisphere, optic radiations, memory-related mesial structures, and deep vascular supply.

The management of unruptured AVMs remains debated, especially after ARUBA, which found better short-term results with medical management over intervention for unruptured AVMs [32]. Nonetheless, later surgical and observational studies have raised concerns about whether ARUBA's findings apply broadly, especially for carefully chosen low-grade AVMs treated at high-volume centers, where microsurgery is often the main treatment [33]. This debate is particularly relevant to temporal AVMs because many cases in the reviewed literature were surgically selected and classified as low- or moderate-grade. Thus, this review's results should not be seen as a universal endorsement for intervention in all temporal AVMs, but as a summary of outcomes mostly derived from surgical treatment cases.

Several limitations should be recognized. Firstly, the evidence mainly comprised retrospective case series, which introduced selection and reporting biases. Secondly, the dataset was heavily weighted toward surgically treated patients, making it difficult to compare equally with embolization, radiosurgery, or conservative treatment. Thirdly, there was notable heterogeneity across various pooled estimates, especially concerning age, seizure presentation, hemorrhage, lesion size, and neurological outcomes. Fourthly, definitions of outcomes varied between studies, and older series often lacked standardized reporting of mRS, GOS, seizure outcomes, and follow-up periods. Lastly, although focusing the analysis on temporal AVMs enhances anatomical specificity, it decreases the sample size and limits subgroup analyses based on lateral versus medial temporal location, rupture status, and treatment approach.

The existing literature indicates that temporal AVMs are most often found in surgically managed groups and are commonly linked to seizures and bleeding. In selected cases-especially those with low to moderate-grade and surgically accessible lesions-surgical intervention tends to achieve high lesion control rates and favorable outcomes. Nonetheless, the current evidence is mainly observational and focused on surgery. To develop more precise management strategies, future multicenter prospective studies should include standardized reporting, extended follow-up periods, and a balanced representation of microsurgery, embolization, radiosurgery, and combined treatments.

## Conclusions

Temporal AVMs often have good obliteration rates and functional outcomes when treated effectively. The literature mainly describes surgical resection as the primary treatment, especially for low- to moderate-grade lesions that are accessible surgically. Factors such as the lesion's location, size, and proximity to eloquent cortex influence treatment choices and outcomes. Adjunct treatments like embolization and radiosurgery are used selectively, typically to complement other interventions. However, most evidence stems from retrospective surgical series, which limits definitive conclusions about optimal management strategies. Future research should include prospective studies with standardized outcome measures and evaluations of multimodal therapies to clarify the role of surgery versus other treatments and to support personalized management approaches.

## Figures and Tables

**Figure 2:**
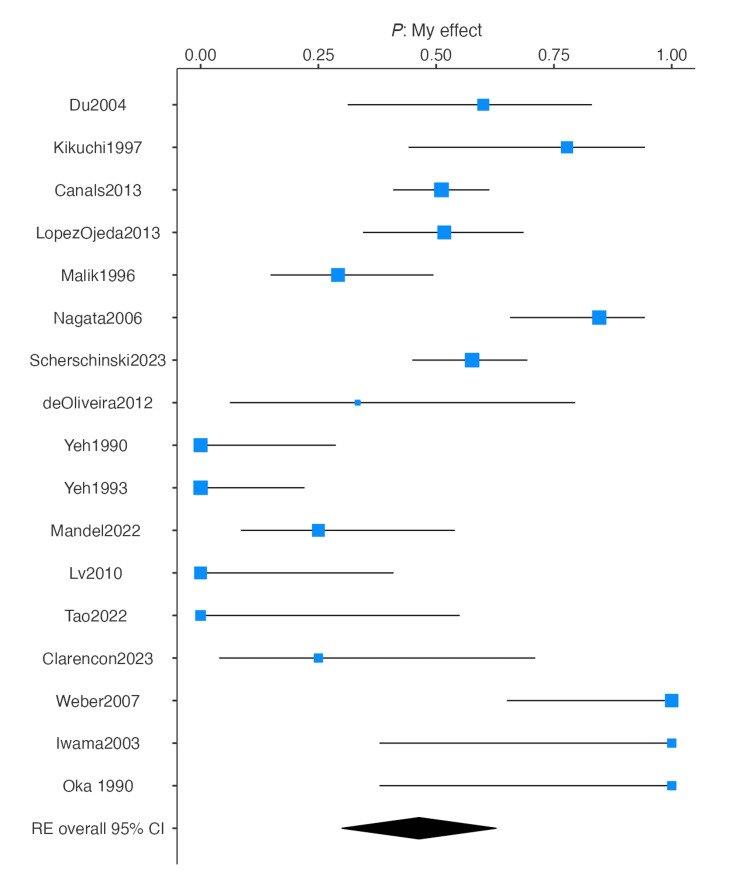
Forest plot showing the proportion of patients presenting with ruptured temporal AVMs. The pooled estimate was 46.4% (95% CI: 30.0% to 62.7%), with high heterogeneity (I² = 92.2%) AVM: arteriovenous malformation; CI: confidence interval

**Figure 3:**
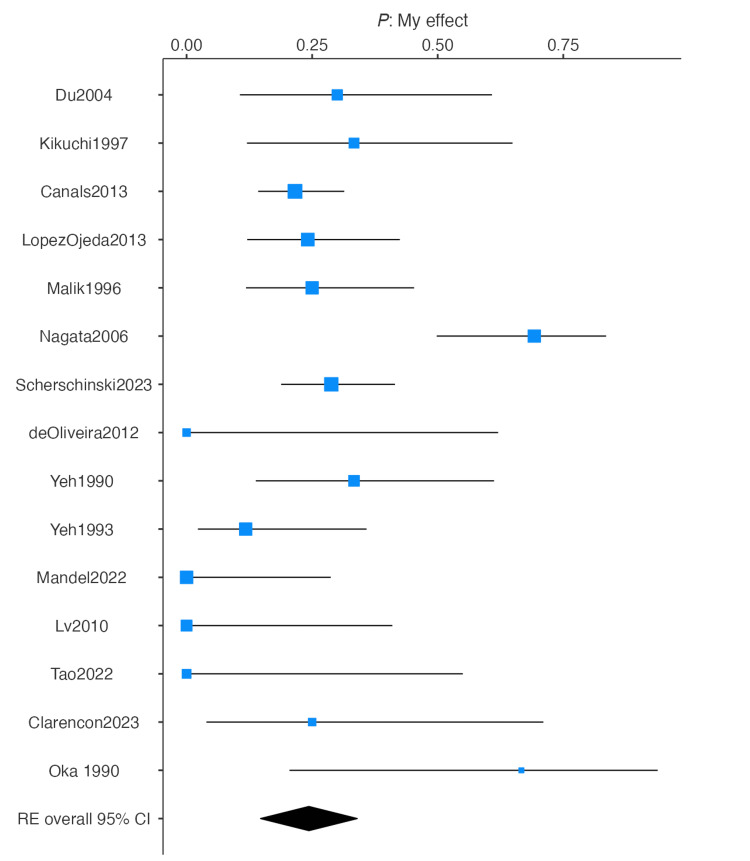
Forest plot showing the overall complication rate following treatment of temporal AVMs. The pooled estimate was 24.4% (95% CI: 14.7% to 34.0%), with substantial heterogeneity (I² = 74.2%) AVM: arteriovenous malformation; CI: confidence interval

**Figure 4:**
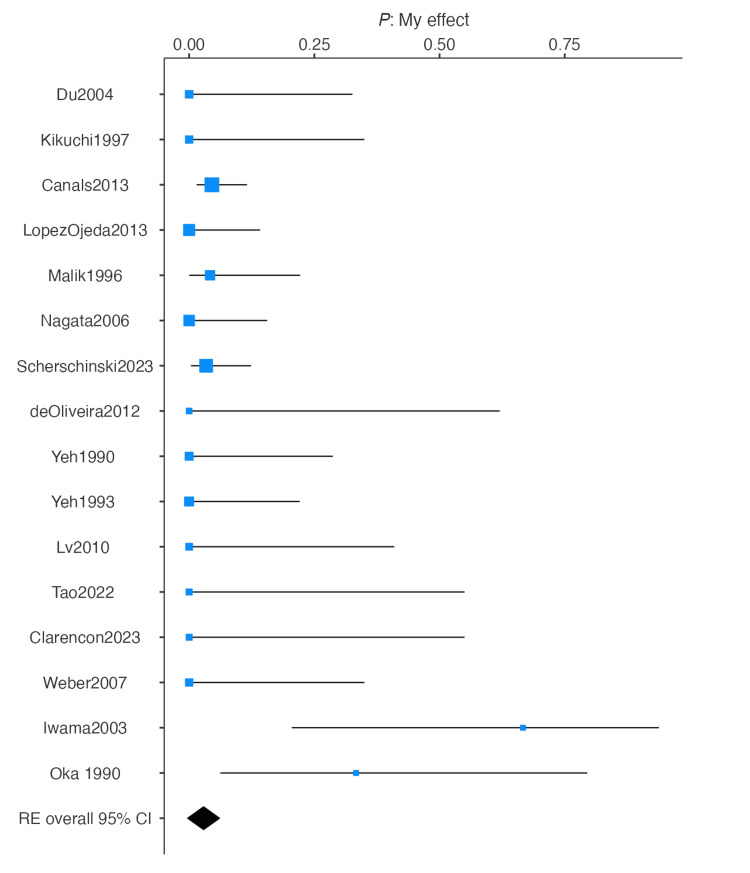
Forest plot showing the pooled mortality rate after treatment of temporal AVMs. The pooled estimate was 2.9% (95% CI: −0.3% to 6.0%), with low heterogeneity (I² = 8.5%) AVM: arteriovenous malformation; CI: confidence interval

**Figure 5:**
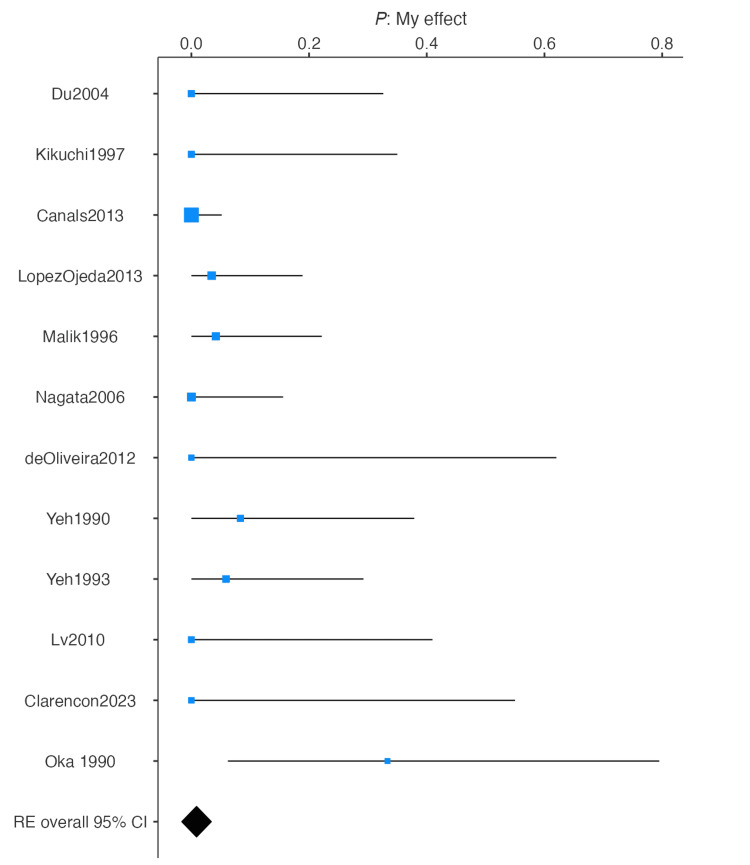
Forest plot showing recurrence after treatment of temporal AVMs. The pooled estimate was 0.9% (95% CI: −1.6% to 3.4%), with no observed heterogeneity (I² = 0%) AVM: arteriovenous malformation; CI: confidence interval

**Figure 6:**
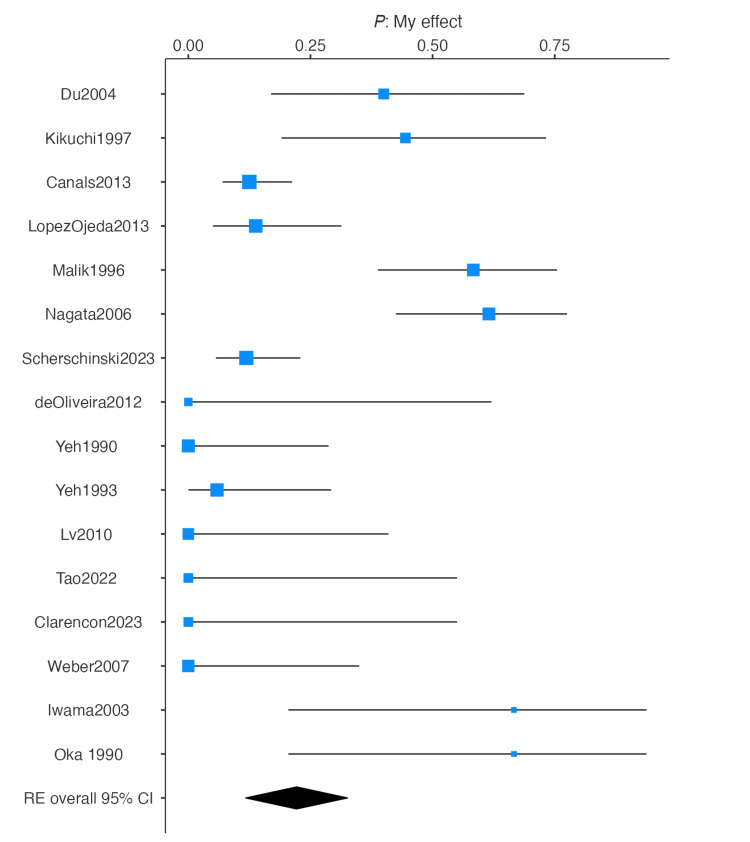
Forest plot showing favorable functional outcome, defined as mRS ≤ 2 or good GOS. The pooled estimate was 76.5% (95% CI: 66.0% to 86.9%), with substantial heterogeneity (I² = 80.8%) mRS: modified Rankin Scale; GOS: Glasgow Outcome Scale; CI: confidence interval
